# Mesenchymal tumours of the mediastinum—part I

**DOI:** 10.1007/s00428-015-1830-8

**Published:** 2015-09-10

**Authors:** Michael A. den Bakker, Alexander Marx, Kiyoshi Mukai, Philipp Ströbel

**Affiliations:** Department of Pathology, Maasstad Ziekenhuis, PO Box 9100, 3007 AC Rotterdam, The Netherlands; Department of Pathology, Erasmus MC, Rotterdam, The Netherlands; Institute of Pathology, University Medical Center Mannheim, University of Heidelberg, Heidelberg, Germany; Department of Diagnostic Pathology, Saiseikai Central Hospital, Tokyo, Japan; Department of Pathology, Universitätsmedizin Göttingen, Göttingen, Germany

**Keywords:** Mediastinum, Mesenchymal tumours, Soft tissue tumours

## Abstract

The mediastinum is an anatomically defined space in which organs and major blood vessels reside with surrounding soft tissue elements. The thymus is an important organ in the mediastinum, and many of the masses encountered in the mediastinum are related to this organ. Most neoplasms diagnosed in the mediastinum are epithelial tumours (thymomas and thymic carcinomas), lymphomas or germ cell tumours. In contrast, soft tissue tumours of the mediastinum are rare. In 1963, Pachter and Lattes systematically reviewed soft tissue pathology of the mediastinum, covering the hitherto described [2, 226, 227] In this review, based on the 2013 WHO classification of soft tissue tumours and the 2015 WHO classification of tumours of the lung, pleura, thymus and heart, we provide an updated overview of mesenchymal tumours that may be encountered in the mediastinum.

## Introduction

Soft tissue tumours arising in the mediastinum are rare. Their estimated incidence is between 2 and 6 % of mediastinal neoplasms [1–3]. However, this often quoted estimate is based on historical series with relatively small numbers of cases. In addition, previous reviews of mediastinal soft tissue tumours often predate novel typing strategies and current classification schemes.

Most soft tissue tumours described elsewhere in the body have been reported to occur in the mediastinum, of course with the exception of strictly site- or organ-specific neoplasms such as GIST. Because of their rarity, most mesenchymal tumours in the mediastinum have been reported as case reports or small series. Mediastinal sarcomas may either arise de novo or rarely as “somatic-type” malignancy in a mediastinal germ cell tumour (GCT). Development of a sarcomatous component has been reported to occur more frequently in mediastinal GCTs than in other sites [4]. The two most common sarcomas developing in mediastinal GCTs are rhabdomyosarcoma and angiosarcoma. In addition, sarcomatous areas as part of a thymic sarcomatoid carcinoma or pseudosarcomatous stroma in a thymoma may sometimes be a diagnostic consideration [5–7]. In this review, we will focus on de novo primary mesenchymal tumours of the mediastinum. Based on the current WHO classification of soft tissue tumours [8], we will systematically review those entities that have been described in the thymus and mediastinum with an emphasis on their site-specific features.

## Adipocytic tumours

Lipomatous tumours are common in the mediastinum and may be located in any compartment. All subtypes of liposarcoma have been reported in the mediastinum.

### Lipoma

Lipoma has been frequently reported in the mediastinum and comprises between 1 and 9 % of primary thymic masses [9, 10]. Mediastinal lipoma may arise from connective tissues both of the mediastinum and the thymus gland itself.

### Thymolipoma

When a circumscribed mediastinal mass lesion is composed of mature fat and has a distinct component of thymic tissue, it is considered a site-specific tumour and is termed thymolipoma [11]. A definite distinction from lipoma that, by definition, is devoid of thymic tissue may not be possible in small biopsies [10, 12–14]. The nature of thymolipoma is unclear. Histogenetic conceptions postulated that (1) thymolipoma is essentially a lipoma (i.e. an adipocytic neoplasm) with incorporation of normal thymic tissue and it is (2) a combined neoplasm of thymic fat and a neoplastic thymic epithelial component, (3) fatty replacement of a thymoma or (4) fatty replacement of hyperplastic thymic tissue (i.e. not strictly a neoplasm) [15].

**Fig. 1:**
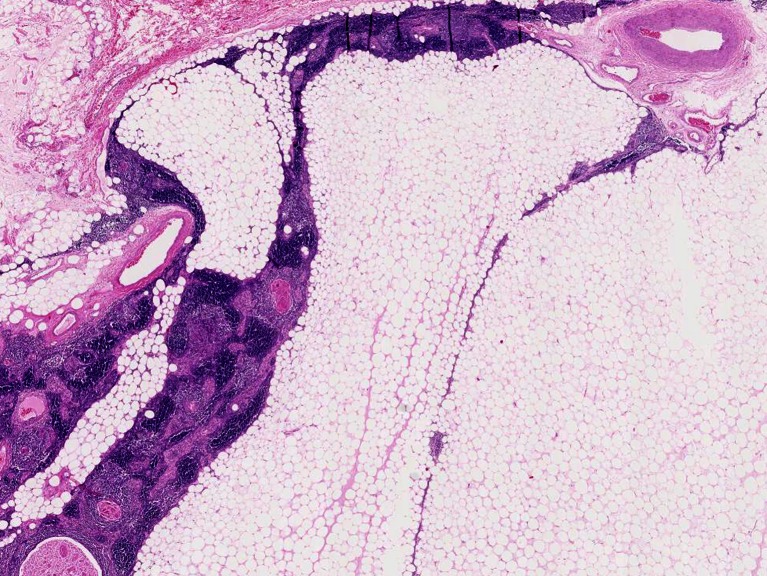
Thymolipoma. Thymolipoma in a 43 year-old male, discovered incidentally during routine physical work-related examination. Mature fat with thymic tissue with discernable cortical and medullary compartments and minor cystic change (HE stain)

**Fig. 2:**
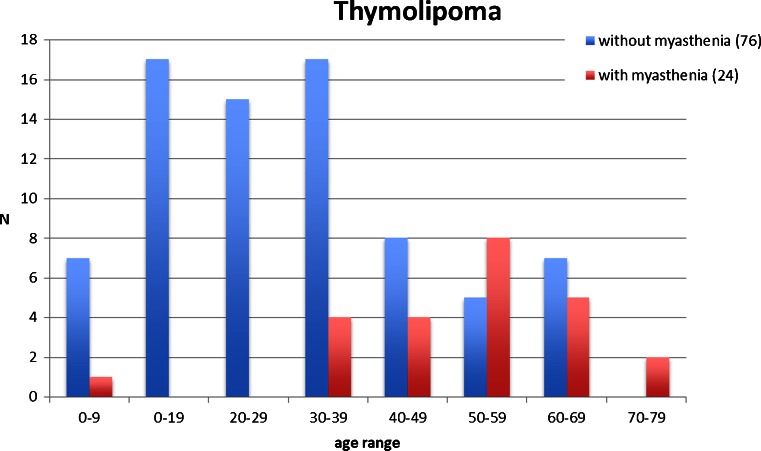
Age distribution of thymolipoma with [21, 23, 24] and without [12, 14, 16, 17] myasthenic symptoms

Thymolipoma (Fig. 1) is mainly seen in young adults in the second to fourth decade but has been described in all ages (range 3–76 years; median 29 years [12, 14, 16, 17]) with several well-documented paediatric cases [18] and with an equal sex ratio. Thymolipoma may reach a large size (up to several kilograms) and is clinically silent in at least one third of cases but may produce symptoms of breathlessness by compression of lung tissue, recurrent infection or pain [14]. Asymptomatic tumours have been known to be present for considerable time, occasionally being confused with cardiomegaly [14, 19–21], although this misperception is unlikely with modern imaging techniques. Thymolipoma may be associated with autoimmune symptoms, such as anaemia, hypogammaglobulinemia, hyperthyroidism [22] and, most frequently, myasthenia gravis (MG). The incidence of MG in thymolipoma varies considerable in series, ranging from 0 to 50 % [12, 14, 15, 17, 22–24], but is less than 5 % in recent larger series [14, 17, 23]. Patients with thymolipoma-associated MG tend to be older, and their tumours tend to be smaller (Fig. 2) [15, 23]. It may well be that MG-associated cases come to medical attention before these tumours reach a large size [22]. Surgical resection is curative, and malignant transformation does not occur.

Histologically, thymolipomas are encapsulated tumours composed of mature fat cells and thymic tissue. The latter consists of epithelial cells and immature TdT-positive lymphocytes (“thymocytes”) and may contain Hassall’s corpuscles. The proportion of fatty tissue may vary considerably (in one series from 30 to 80 %) [14, 17]. Rare occurrence of a thymoma and even thymic carcinoma arising in thymolipoma has been reported [25, 26].

A number of unusual variants of thymolipoma have been described, including one with a prominent vascular component, designated thymohemangiolipoma [27]. However, fatty change is a well-known alteration in soft tissue hemangioma and arterio-venous vascular malformations, and the described tumour could therefore also be considered a thymic vascular tumour with lipomatous stroma. Other variants of thymolipoma contained striated skeletal muscle (“myoid”) cells, similar to the rare myoid cells which occur in the normal thymus [28–30]. Two cases of thymolipoma (one in a 9-year-old girl) with areas of fibrocollagenous tissue were designated as “fibrothymolipoma” [31].

### Lipomatosis

Although lipomatosis is considered a non-neoplastic increase of normal mature fat, this non-encapsulated mass lesion is mentioned here for differential diagnostic purposes. In the mediastinum, it is usually detected by imaging studies, where it causes widening of the mediastinum. Patients may complain of dyspnea. It is commonly associated with steroid use, Cushing’s disease or obesity. Idiopathic cases are very rare [32–34].

### Lipoblastoma

Lipoblastoma (LPB) is a rare adipocytic tumour composed of fat cells in various stages of maturation, essentially restricted to the paediatric age group with 90 % occurring before 3 years of age without sex predilection. Mediastinal LPB is very rare with approximately 30 cases presented in case reports or small case series [35–66]. Mediastinal LPB may grow to a large size and pack a large volume of the thorax, and the precise origin of the tumour may be difficult to determine in these cases. A significant number of mediastinal LPB extend into the neck. In the series of mediastinal LPB, patients ranged from 6 months to 6.5 years of age (median 19.5 months).

Histologically, LPB is characterized by fatty tissue in varying degrees of maturation. Most cases are circumscribed, but rarely, a diffuse growth is seen (diffuse LPB, lipoblastomatosis). Although LPBs are benign tumours, recurrence may occur [35] and the tumours may envelop vital structures or extend within orifices such as the spinal canal, hampering complete removal [43, 51, 58].

### Lipoma variants

**Fig. 3:**
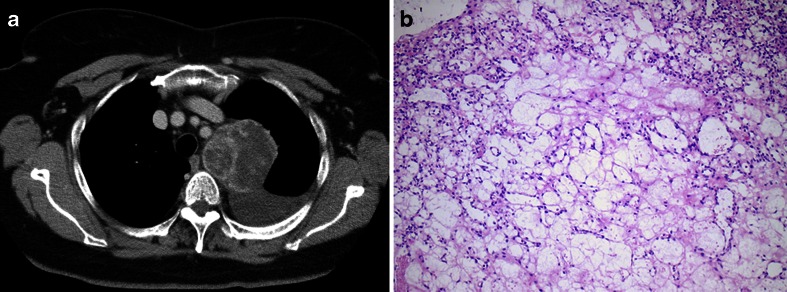
Angiolipoma (lipomatous hemangioma). Tumour in the superior mediastinum in a 67-year-old female who had few complaints but had persistent pleural effusion. The mass was excised, and no recurrence was noted after several years of follow-up. **a** Axial contrast-enhanced CT image revealing a circumscribed mediastinal mass with variable density. The mass was found to have been present for several years on retrospective evaluation of previous examinations. **b** Mature fat admixed with thin-walled vessels; there was no atypia of endothelial cells

Angiolipoma, an adipocytic tumour composed of small, often capillary-sized blood vessels, combined with mature fat cells has been described in the mediastinum (Fig. 3). As the proportion of fat and blood vessels may vary considerably from case to case, it is debatable whether some of these cases could also be considered primary vascular proliferations (capillary hemangiomas) with an adipose component [67–71].

A few cases of mediastinal spindle cell lipoma with typical histological features including “ropey” dense collagen strands and bland spindle cells have been reported [72, 73]. The immunohistochemical profile with CD34 positivity supported the diagnosis in these cases, but there have been no reports documenting the presence of 16q or 13q chromosomal aberrations that are characteristic of peripheral spindle cell and pleomorphic lipomas [69].

Hibernoma is a rare distinctive benign adipocytic neoplasm composed of brown fat with microvesicular multivacuolated lipocytes, usually in a subpleural site [74] or associated with soft tissue of the chest or extending from the neck [75]. Only very few cases of hibernoma have been reported as mediastinal masses [76–79].

Less than ten cases of myelolipoma have been reported in the mediastinum. Most cases of myelolipoma occurred in adults (sixth to eighth decade) with no gender predilection and were often discovered incidentally or at autopsy where death was due to other causes. Most cases were present in the posterior mediastinum, often in a paravertebral location [69, 80–89]. Similar to in the adrenal gland, the most common site of myelolipoma, hematopoietic tissue and mature fat are combined in varying proportions. In patients with hematologic disease, similar masses can occur as either normal extramedullary hemopoiesis or as extra-medullary extension of the hematologic tumour itself and should not be considered myelolipoma [90].

### Liposarcoma

Liposarcoma is a malignant adipocytic tumour with various subtypes which impact on behaviour and prognosis. It is, by far, the most common primary malignant mesenchymal tumour of the mediastinum. The age range is wide. Paediatric cases, even occurring in very young infants, have been reported [16, 91–98]. Although an origin from thymic tissue has been shown in a minority of cases (“thymoliposarcoma”) [93, 95, 99], cases have also been documented in the posterior mediastinum [93, 100–102], suggesting a non-thymic origin. Although numerous case reports and several series [16, 93, 95, 101, 103, 104] of mediastinal liposarcoma have been published, most of these predate the current molecular insights of liposarcoma which may aid in classification. In general, mediastinal liposarcomas are large, and tumours up to 7 kg have been reported [105]. Tumours may remain asymptomatic for long periods. Shortness of breath and pain are the most common symptoms. Despite their large size, vena cava syndrome has only rarely been reported [92, 102].

All subtypes of liposarcoma have been reported in the mediastinum. Based on their own series and a comprehensive review of 142 published cases, Boland et al. concluded that the proportions of liposarcoma subtypes in the mediastinum differ from those in other sites [106]. In particular, pleomorphic liposarcoma was considerably more prevalent in the mediastinum. In addition, mediastinal liposarcomas, including the pleomorphic subtype, frequently contained myxoid areas, a finding which has been confirmed in other series [93, 101]. Although the prevalence of mediastinal myxoid liposarcoma was approximately similar to other anatomic locations, a round cell component was not identified in the mediastinal tumours. This was confirmed in other series, in which the presence of a round-cell component was exceptional [93]. The molecular signatures of mediastinal liposarcomas correspond to those of liposarcomas elsewhere, with presence of the t(12;16) *FUS-DDIT-3* fusion in myxoid liposarcoma [107] and amplification of *mdm-2* in well-differentiated and dedifferentiated liposarcoma [101, 108].

**Fig. 4:**
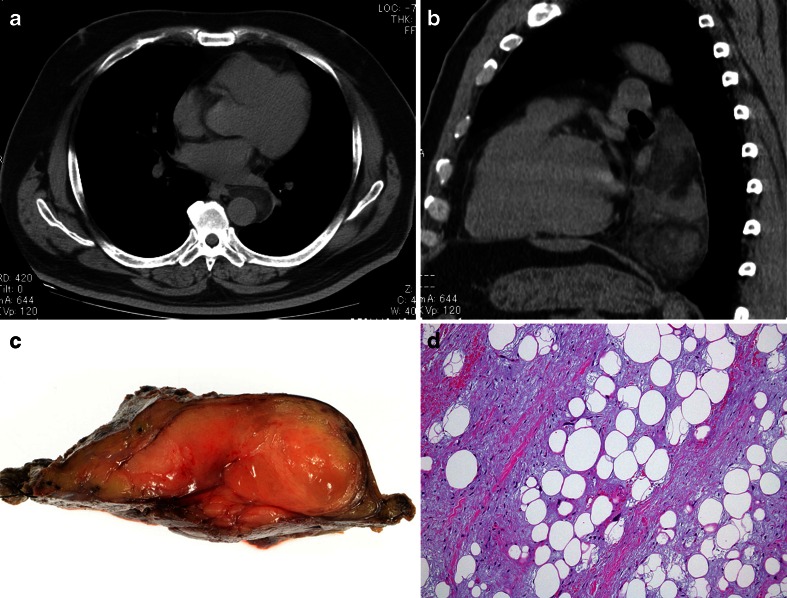
Liposarcoma with myxoid change. A posterior mediastinal mass, surrounding the aorta in a 55-year-old male discovered during routine health check (**a**). The mass was excised (**b**, **c**). A recurrence was excised 16 months later; there was no evidence of disease after 1-year follow-up. **a** Axial noncontrast CT image at the level of the left atrium revealing a smoothly marginated retrocardiac mass with variable attenuation. **b** Sagittal reformatted CT image showing the retrocardiac mass to extent from the aortic arch to the diaphragm. **c** Macroscopic image of the transected tumour revealing a fleshy, partially thinly encapsulated mass. **d** Microscopic image (medium power, HE stain) showing myxocollagenous stroma with scattered atypical mesenchymal cells

An unusual liposarcoma, termed pleomorphic myxoid liposarcoma (P-MLPS) by Alaggio et al., appears to have a predilection for the mediastinum of young patients [103] (Fig. 4). Five of the 12 cases in their series arose in the mediastinum in patients aged from 13 to 20 years of age. Rare adult cases were described in the series of Boland et al. [106]. In addition to areas similar to conventional low-grade myxoid liposarcoma, foci with increased cellularity, hyperchromatic cells and pleomorphic lipoblasts with increased mitotic activity were observed in areas with loss of the typical capillary vascular pattern and necrosis. These tumours followed an aggressive course, with death occurring within 3 years of diagnosis in three of five patients with available follow-up data. Neither a t(12;16)(q13;p11), the characteristic genetic hallmark of myxoid liposarcoma, nor amplification of *mdm*-2, a marker for well-differentiated and dedifferentiated liposarcoma, was identified. Other unusual variants of liposarcoma that have been described in the mediastinum contained elements of smooth muscle (lipoleiomyosarcoma) or skeletal muscle [101, 109–111].

The main differential diagnostic considerations of mediastinal liposarcoma are benign lipomatous tumours. Attention to histological features and the use of molecular markers will lead to a correct diagnosis. Dedifferentiated liposarcoma may mimic undifferentiated pleomorphic sarcoma, particularly in biopsy specimens; demonstration of amplification of *mdm*-2 confirms the diagnosis of liposarcoma. So-called fat-forming solitary fibrous tumour may enter the differential diagnosis of liposarcoma. *Mdm*-2 amplification is absent in these tumours, and positive staining of STAT-6 on immunohistochemistry may further support a diagnosis of solitary fibrous tumour (SFT) (see below). Liposarcoma as a secondary malignancy developing in a GCT has very rarely been described [112]. Curative surgical resection is the treatment of choice. The mortality of mediastinal liposarcomas ranges from 30 to 50 %.

## Fibroblastic/myofibroblastic tumours

Since many of the tumours in the fibroblastic/myofibroblastic category have a predilection for the skin and superficial soft tissues, it is not surprising that only very few of the entities in this category have been reported in the mediastinum. Desmoid tumours (aggressive fibromatosis), SFT and inflammatory myofibroblastic tumour (IMT) are among the more frequently reported types in the mediastinum.

### Aggressive fibromatosis /desmoid tumour

Aggressive fibromatosis is a locally invasive (myo)fibroblastic proliferation with bland cytology and without metastatic potential. About 30 cases of primary mediastinal aggressive fibromatosis (AF) have been reported as case reports, many of which were included in reviews by Nakagiri et al. and Bouchikh et al. [113, 114] AF in the mediastinum occurs mainly in younger individuals (age range 3–67 years; median 38 years) [113–119]. Some cases were associated with surgical scars [114]. There are no published data about an association with familial adenomatous polyposis (FAP) or Gardner syndrome. In contrast to non-mediastinal AF, mediastinal AF cases appear to occur slightly more frequently in males [120]. Similar to non-mediastinal AF, mediastinal recurrence is common after surgical removal, which, in this location, is hampered by anatomical constraints. Unresectable AF in the mediastinum may be fatal.

### Solitary fibrous tumour

SFT is an uncommon but well-known intrathoracic fibroblastic tumour and often of pleural origin. Since its description, numerous reports of extra-pleural SFT cases have been published with tumours arising in almost all locations in the body [121]. Mediastinal SFTs without a clear connection with the mediastinal pleura have been described in case reports and small series. Hemangiopericytoma, which has also been described in the mediastinum, is now considered identical to SFT.

Mediastinal SFT may arise in any compartment of the mediastinum. A number of mediastinal SFTs had also contact to the epicardium [122], and in such cases, it may be questionable whether these represent true primary mediastinal SFT [123–125]. Thoracic SFTs reach a large size and may extend to the chest wall, protrude in the lung or even invade other structures [124]. SFTs occur over a wide age range but are typically seen in older adults [126–128]. Paediatric cases are very rare [129–131]. There is no sex predilection [122–124, 129–169].

The histomorphology of SFT is characterized by a “patternless” architecture with randomly distributed hypocellular and hypercellular areas, sometimes keloid-like collagen, and thin “stag-horn” branching capillary vessels that may show perivascular hyalinization. Tumour cells are generally spindle shaped with bland cytology and few mitoses. Immunohistochemistry is helpful in the diagnosis of SFT and shows usually strong expression of CD34 [121, 166]. Other markers typically positive in SFT are CD99 and Bcl2 [127]. Variable staining is seen with smooth muscle actin, while cytokeratin positivity is very rare [163], except in variants with epithelioid morphology (see below). Recently, nuclear staining of STAT6 resulting from *NAB2-STAT6* gene fusion [170–172] has been shown to be a more specific marker for SFT [173–175], although presence of the *NAB2-STAT6* gene fusion has not yet been formally shown for mediastinal SFT.

It has been suggested that mediastinal SFTs more frequently show aggressive behaviour compared to non-mediastinal SFTs [169]. The same criteria predicting malignancy, particularly a high mitotic count (>4 mitoses per 2 mm^2^), high cellularity, pleomorphism and necrosis apply as outside the mediastinum [127].

**Fig. 5:**
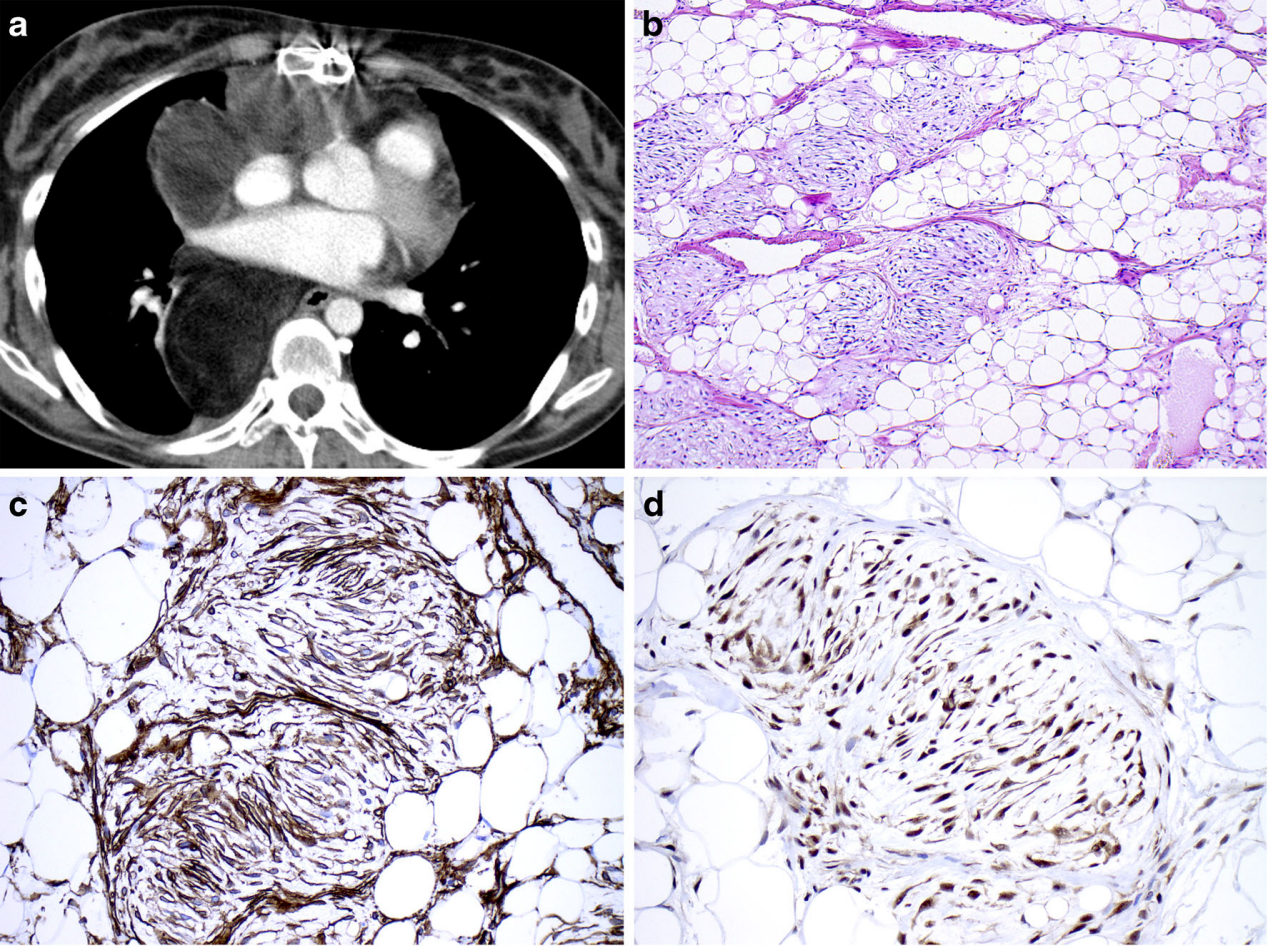
Fat-forming solitary fibrous tumour. A large tumour in the chest cavity in a 23-year-old female who complained of shortness of breath. The mass was excised but later recurred and resulted in death of the patient. **a** CT image showing a large tumour that occupies the anterior and posterior mediastinum. **b** HE stain showing mature fat tissue admixed with ill-defined nodules of spindle cells with elongated nuclei and tapering cytoplasm. **c** CD34 stain which is diffusely positive in the spindle cells. **d** STAT-6 immunohistochemistry with diffuse nuclear and cytoplasmic positivity

So-called fat-forming SFT shows adipocytic differentiation to such an extent that well-differentiated liposarcoma may enter the differential diagnosis [132, 143, 151, 176] Although nuclear STAT6 expression has been shown to be a useful marker for the diagnosis of fat-forming SFT, particularly in CD34 negative cases [177] this has also been demonstrated in a significant proportion of dedifferentiated liposarcomas [108, 178]. However, staining was limited to the non-lipogenic sarcomatous areas. In addition, *mdm-2* amplification remains a robust marker for liposarcoma and has not been reported in SFT (Fig. 5).

Other variants of SFT include cases with an epithelioid morphology, either as a focal component in combination with “classical” spindle cells or as tumours that may be exclusively epithelioid [153, 179–185]. This variant may stain for cytokeratin, which may also be seen in the spindle cells. A mediastinal epithelioid SFT was reported by Marchevsky et al., who considered the differential diagnosis of adenomatoid tumour [152]. Epithelioid areas in the highly unusual thymic SFT case reported by Tsubochi et al. showed glandular, neuroepithelial and neuroendocrine morphology in addition to classical SFT features [164]. Immunohistochemistry reflected the diverse histology with STAT6 staining restricted to the classical SFT component.

Mediastinal SFT, in particular malignant cases, may be confused with thymomas with a prominent spindle cell morphology (e.g. type A thymoma), mesothelioma, sarcomatoid carcinoma, synovial sarcoma and malignant peripheral nerve sheath tumour (MPNST). In addition to morphological differences, immunohistochemistry will help to diagnose SFT in most cases. Cytokeratin staining is seen in thymoma, mesothelioma, sarcomatoid carcinoma and synovial sarcoma and is very rare in SFT. In addition, STAT6 is a reliable marker for SFT and has not been described in the other tumours.

### Inflammatory myofibroblastic tumour

Inflammatory myofibroblastic tumour (IMT), a tumour composed of (myo)fibroblastic cells and a variably dense non-neoplastic inflammatory component, has been reported in many sites, predominantly in young adults and children [186]. Bona fide mediastinal IMT is rare with less than 20 convincing cases reported in the English literature [186–196]. Similar to IMT in general, which is predominantly a tumour of children and young adults [186], mediastinal IMT appears to arise mostly in young adults (range 13–72; median age 34) with a slight female predominance (M/F = 5:8) [187, 188, 190, 192–196].

Histologically, IMT is an infiltrative tumour composed of (myo)fibroblastic cells admixed with an inflammatory infiltrate of lymphocytes, plasma cells and eosinophils. Three histological patterns (granulation tissue-like/compact fascicular spindle cell pattern with marked inflammation/low cellularity scar-like pattern) that can all occur within a single tumour have been described but do not portend a specific biological behaviour [186].

Multifocality is not uncommon in IMT. Cytoplasmic expression of ALK protein is detectable in about half of cases and correlates well with presence of *ALK* gene rearrangements. *ALK* aberrations have been reported in a single mediastinal case [192].

The biological behaviour of IMT is variable and recurrences are common, but distant metastasis is rare.

Similar to non-mediastinal sites, treatment rests mainly on complete local excision. Patients with *ALK*-rearranged IMT are potential candidates for treatment with specific inhibitors, although this has so far not been described for mediastinal cases.

### Other fibroblastic/myofibroblastic tumours

Calcifying fibrous pseudotumour, a very rare soft tissue tumour with proposed but unconfirmed relationship to IMT, has been reported in the mediastinum [197–200]. The tumours all occurred in the mediastinum of adults. No evidence of disease was recorded during 11–49-month follow-up after surgery in three patients.

Reports of fibrosarcoma arising in the mediastinum predate the current concept that adult fibrosarcoma is a very rare tumour. The most recent report of an adult mediastinal fibrosarcoma dates from 1995 [201]. While in older reports, fibrosarcoma was one of the more common reported soft tissue tumours, it is likely that these would now be diagnosed as dedifferentiated liposarcoma, MPNST or synovial sarcoma. Infantile fibrosarcoma has not been reported in the mediastinum.

Three cases of low-grade fibromyxoid sarcoma have been reported in the mediastinum, among which one case was attached to the epicardium [202–204]. All cases occurred in middle-aged patients, and one patient had a late recurrence 9 years following surgery, which was resected; metastases were not recorded.

A single case of infantile myofibromatosis with multifocal masses in the lung and mediastinal soft tissue in a 4-week-old male infant has been reported. Five years after surgical resection and pneumonectomy, no disease activity was recorded [205].

A single case of mediastinal giant cell angiofibroma was reported in a 62-year-old female [206]. However, giant cell angiofibroma, which is most commonly described in the orbit, is now considered a variant of SFT [207].

### Fibrohistiocytic tumours

Fibrohistiocytic tumours are mainly encountered in superficial soft tissues or joints. Two cases of benign fibrous histiocytoma in the mediastinum have been reported [208, 209]. Despite its terminology, angiomatoid fibrous histiocytoma is commonly included among tumours of uncertain origin and is not considered a variant of benign fibrous histiocytoma. Two cases of angiomatoid fibrous histiocytoma have been reported in the mediastinum [210, 211]. Both tumours were resected without evidence of recurrence or metastases.

Giant cell tumour of soft tissue (GCT) is most commonly observed in superficial soft tissue of the extremities, trunk and head and neck. Five primary mediastinal cases have been reported, occurring in individuals aged 18–53 years (in three female and two male patients) [212–214]. The tumours were almost all located in the posterior mediastinum and ranged from 2.5 to 15 cm, filling the hemithorax. No recurrences were noted, but the follow-up period was not stated in all published cases.

Malignant fibrohistiocytic tumours, previously referred to as malignant fibrous histiocytoma (MFH), are now classified as undifferentiated high-grade pleomorphic sarcomas and are not considered of true histiocytic lineage (see part 2 of this review). So-called MFH cases have been recorded in the mediastinum with generally poor prognosis.

## Secondary mediastinal mesenchymal tumours in germ cell tumours

When a mediastinal sarcoma is diagnosed, the possibility must be considered that sarcomatous tissue remains as the sole component in a long-standing primary mediastinal GCT, particularly in younger male patients [215]. Likewise, a mediastinal sarcoma could be the metastatic vestige of a burnt-out primary gonadal GCT. Demonstration of a germ cell (GC) origin of a mediastinal sarcoma may be accomplished by thorough sampling to reveal GCT remnants. Serological investigations (β-HCG, AFP) may reveal a cryptic GCT. The presence of isochromosome 12p (i(12p)) is a characteristic abnormality of type II GCTs [216, 217] which persists in the somatic elements when the typical GCT components are no longer recognizable [218–220]. Thus, identifying an i12p in the mesenchymal tissue could confirm the origin of a mediastinal sarcoma from a GCT. Development of a sarcomatous component in a GCT occurs more frequently in mediastinal GCTs than in other sites [4]. It has been suggested that this results from the fact that GCTs in this location grow undetected over longer time periods, are larger and may acquire malignant somatic tumour subclones more frequently [215]. The two most common sarcomas developing in mediastinal GCTs are rhabdomyosarcoma and angiosarcoma [4, 215, 221, 222]. Less commonly reported sarcomas arising in GCTs are leiomyosarcoma, MPNST and, rarely, liposarcoma [4, 215, 222, 223]. Although most secondary sarcomas in GCTs develop in the anterior mediastinum, rare cases arise in the posterior mediastinum. Similarly and in analogy to somatic (teratomatous) elements becoming the dominant tissue in GCTs treated by chemotherapy, sarcomatous tissue may be the only residual tissue left after chemotherapeutically treated GCTs [224, 225]. Treatment should be tailored to the somatic component rather than to its germ cell origin [223]. Nevertheless, similar to non-germ-cell-related sarcoma, the prognosis of a mediastinal sarcoma arising in a GCT is poor.
